# Grouping sounds into evolving units for the purpose of historical language comparison

**DOI:** 10.12688/openreseurope.16839.2

**Published:** 2024-08-20

**Authors:** Johann-Mattis List, Nathan W. Hill, Frederic Blum, Cristian Juárez

**Affiliations:** 1 Chair of Multilingual Computational Linguistics, University of Passau, Passau, Bavaria, 94032, Germany; 2 Department of Linguistic and Cultural Evolution, Max Planck Institute for Evolutionary Anthropology, Leipzig, Saxonia, 04103, Germany; 3 Trinity Centre for Asian Studies, The University of Dublin Trinity College, Dublin, Leinster, Ireland

**Keywords:** historical language comparison, phonetic transcription, representation of speech sounds

## Abstract

Computer-assisted approaches to historical language comparison have made great progress during the past two decades. Scholars can now routinely use computational tools to annotate cognate sets, align words, and search for regularly recurring sound correspondences. However, computational approaches still suffer from a very rigid sequence model of the form part of the linguistic sign, in which words and morphemes are segmented into fixed sound units which cannot be modified. In order to bring the representation of sound sequences in computational historical linguistics closer to the research practice of scholars who apply the traditional comparative method, we introduce improved sound sequence representations in which individual sound segments can be grouped into evolving sound units in order to capture language-specific sound laws more efficiently. We illustrate the usefulness of this enhanced representation of sound sequences in concrete examples and complement it by providing a small software library that allows scholars to convert their data from forms segmented into sound units to forms segmented into evolving sound units and vice versa.

## Plain language summary

In linguistics, it is difficult to clearly draw the boundaries between the sounds in individual words. What one linguist may analyze as two sounds, another linguist might analyze at just one sound. Since the segmentation of words into sounds is crucial for many analyses in linguistics and since no perfect solution can be found, we offer a new representation that allows scholars to analyze the sounds in a word in a more flexible way that conforms to general standards while at the same time giving linguists enough flexibility to advance individual analyses.

## Introduction

Over the last two decades an ever-increasing number of publications have applied quantitative approaches in historical linguistics. While early work focused almost exclusively on phylogenetic approaches, using manually annotated cognate sets to automatically infer the most plausible phylogenies for the divergence of language families ( Chang *et al.*, 2015; Gray & Atkinson, 2003), more recent research broadens this trajectory of inquiry in three directions. Some recent work concentrates on the standardization and the concrete representation of cross-linguistic data ( Crist, 2005; Forkel *et al.*, 2018; Hill & List, 2017), some studies try to develop workflows that automate sub-steps of the traditional comparative method ( Jäger, 2013; Kondrak, 2000; Prokić *et al.*, 2009; Steiner *et al.*, 2011; Wu *et al.*, 2020), and an even smaller amount of research tries to make active use of tools for symbolic computing in order to implement models of sound change ( Hartmann, 2003; List, 2024a; Marr & Mortensen, 2024).

One of the most contested aspects of all three new research venues in computational historical linguistics is the representation of the form part of a linguistic sign as a sequence of sounds. Although the linear aspect of the linguistic form has long since been emphasized in the linguistic literature ( de Saussure, 1916), and although all sound laws are essentially based on the sequential representation of words and morphemes, practitioners of the comparative method as well as phonologists are usually unsatisfied when computer programs represent a word as a sequence of sounds, pointing to the continuity of the sound signal or the arbitrariness of assigning overlapping articulatory gestures to discrete sound units. With this brief report, we try to propose a solution for the problems resulting from strict word segmentation in historical language comparison, by offering a novel methodology to represent, annotate and compare sound units that do not necessarily consist of individual sounds. We show how our approach can be applied in the comparison of data from different language families.

## Background

Scholars often emphasize the arbitrariness of segmenting speech into distinct sounds. Since the speech signal is a continuum it is indeed not always straightforward to determine a cut-off point in an objective manner. The problem of segmentation is also important for the level of phonetic transcription ( Round, 2023). When dealing with a word like German *Apfel* “apple”, for example, one must decide if one wants to treat the sounds [ p] and [ f] as one affricate sound [ pf] or two distinct sounds. While there are ways to justify the affricate reading in synchrony for morphological reasons, the major diachronic reason for treating the *pf* in German *Apfel* as an affricate is that [ pf] goes back to earlier [ p]. The sound [ pf] in German has thus evolved as one unit, and it keeps evolving as such.

While the case of the labiodental affricate in German may be considered as uncontroversial, there are certain sound combinations which are typically treated as separate sounds in synchronic phonology, which would be better treated as one evolving unit from a historical viewpoint. Consider, for example, sound sequences like [ s t], [ s p], [ s k], [ s m], [ s n], [ s l], and [ s r] occurring as syllable onset in Indo-European languages. While these are typically treated as two distinct sounds, they tend to show very similar sound change patterns in particular Indo-European languages. In German, for example, the alveolar sibilant [ s] tends to become a post-alveolar sibilant [ ʃ], while — with exception of [ k] — the following sound is not only unchanged, but also resists certain sound change patterns, like Grimm's law ( Grimm, 1822), which would be active otherwise. Instead of treating these changes as distinct sound laws, such as

(1) **s > ʃ / _ [p t k m n l r]**,

and

(2) **[p t k m n l r] > [p t – m n l r] / s _**,

one could use a single sound law that captures these cases directly:

(3) **s [p t k m n l r] > ʃ [p t – m n l r]**.

Note that such a representation does not automatically mean that the sound law represents the actual sound change processes more truthfully. Especially in the case of the change of [ s] becoming [ ʃ] in German, German orthography, which represents the [ ʃ] going back to [ s] followed by [ m n l r] as *sch*, while [ s] followed by a plosive is still rendered as *s*, gives us some hints that the sound change processes may have happened at different times in the history of the language ( von Polenz, 2021: 178f).

However, even if it may not always seem justified to treat a certain sound sequence as one single sound unit in a given language family, it can be very practical — with respect to the formulation of sound laws — to cluster sounds into units which are known to evolve together.

This practice of clustering sounds into evolving units has been routinely used in studies on South-East Asian languages, where the rigid syllable structure of many languages makes it much easier to consider sound laws for syllable onsets contrasted with syllable rhymes than to break these further down to sound laws occurring with initials versus medials versus nucleus vowels and codas (see, for example, Ratliff, 2010 for Hmong-Mien languages, Jacques, 2021 for Hmongic languages, or Sprigg, 1972 for Tibetic languages).

## Grouping sounds into evolving units

So far, computational approaches to historical language comparison have represented words and morphemes as rigid sequences of individual sound units whose segmentation cannot be further modified. The strictness is mainly justified by the fact that computational approaches have difficulties to recognize valid sounds when the segmentation is leveraged. Thus, while a software package like LingPy ( List & Forkel, 2023, https://pypi.org/project/lingpy) can recognize a large number of symbols and symbol combinations from the IPA and similar phonetic transcription systems, the software needs to process these symbols in isolation. If symbols were parsed in combination, a specific algorithm would be required to recognize meaningful sub-units in order to understand their major sound properties, which are crucial for the application of phonetic alignment analyses and cognate detection routines ( List, 2014). Similarly, while reference catalogs like the Cross-Linguistic Transcription Systems (CLTS, https://clts.clld.org, List *et al.*, 2024) offer detailed feature descriptions of an abundance of possible speech sounds (currently more than 8000 sounds are attested in cross-linguistic datasets), they do not account for the combinations of sounds into larger units.

Although it is very likely that the number of distinct speech sounds accounted for by the CLTS reference catalog is too large to reflect the linguistic reality of phonetic diversity in the languages of the world (see Rubehn *et al.*, 2024 for experiments to reduce the CLTS feature system in a systematic manner), the fact that more than 8000 distinct sounds that one would not traditionally treat as clusters of smaller sound units can be generated by a system that is based on distinctive features shows that it would not be feasible to try to account for all possible sound combinations that one can observe in different languages.

But since the clustering of distinct speech sounds into larger units reflects an important practice in historical linguistics — which was already discussed by ( Grimm, 1822: 590), who emphasized that exceptions of his *Lautverschiebung* were due to their clustering with the spirant *s-* — we consider it important to allow for the individual, expert-informed grouping of sounds in the representation of sound sequences. Our proposal is therefore to leverage the strict requirement of segmenting the linguistic form into distinct sound units while at the same time preserving the information on distinct sounds in a given dataset. We achieve this goal by adding more flexibility in the representation of sound units without sacrificing the original level of segmentation required by reference catalogs and software for automated sequence comparison.

### Annotation

Our proposal is very straightforward. While the current representation of sound sequences uses a space character as a segmentation symbol, we add the dot character (<.>) as an additional symbol that allows for the combination of sounds into units which would otherwise be segmented. For example, when dealing with a sound sequence like Chinese *quán* 全 “complete” [ tɕʰ ɥ ɛ n ^35^ ], we can “desegmentise” the sequence by grouping the sounds as [ tɕʰ.ɥ ɛ.n ^35^ ] and effectively treating the initial and the medial as one segment as well as the nucleus vowel and the final consonant.

The advantage of this representation — at least for the case of Chinese and many South-East Asian languages with a similarly restricted syllable structure — becomes immediately evident when comparing phonetic alignments of the data. In Table 1, we contrast the “traditional” alignment, as it has been carried out so far in most applications (see e.g., List, 2014), with our new alignment where we cluster sounds historically likely relevant units, which means in the case of the Chinese dialects to assign initials and medials to a common onset group while merging nucleus vowel and coda into a common rhyme group (data taken from Liu *et al.*, 2007, as prepared in Wu and List, 2023).

**Table 1.** Phonetic alignments of four words for “even” in Chinese dialects in segmented and “desegmented” form. On the left, the full alignment with three gapped sites is shown (cells with a – symbol shaded in gray). On the right, the words have been segmented into initial, final, and tone, and the resulting alignment has no gapped sites.

Variety	Alignment						Variety	Alignment		
Bějīng	pʰ	-	i	ŋ	³⁵		Bějīng	pʰ	i.ŋ	³⁵
Wēnzhōu	b	-	e	ŋ	³⁴¹		Wēnzhōu	b	e.ŋ	³⁴¹
Xiàmén	p	j	æ	-	²⁴		Xiàmén	p.j	æ	²⁴
Méixiàn	pʰ	j	a	ŋ	¹¹		Méixiàn	pʰ.j	a.ŋ	¹¹

What can be seen from the example is that this new annotation — in which we conjoin certain segments in our standardized sound sequences into larger units — allows us to align the data without the usage of gap symbols (-). Reducing gaps in phonetic alignments has two major advantages. First, since gaps often depend on the context in which they occur (compare the gap for the coda in Xiàmén our example, which appears because this dialect has dropped certain nasals following the main vowel, most likely via a stage in which the vowel was nasalized), clustering sounds into groups helps us to show the underlying processes in a much more integrated way, rather than proposing the loss of one sound in a specific context. Second, since gaps in phonetic alignments can be rarely interpreted as the loss or gain of an entire sound during sound change, avoiding gaps in our alignments helps us to bring the underlying processes that can be inferred from the alignments much closer to linguistic theory.

### Representation

**Figure 1.**
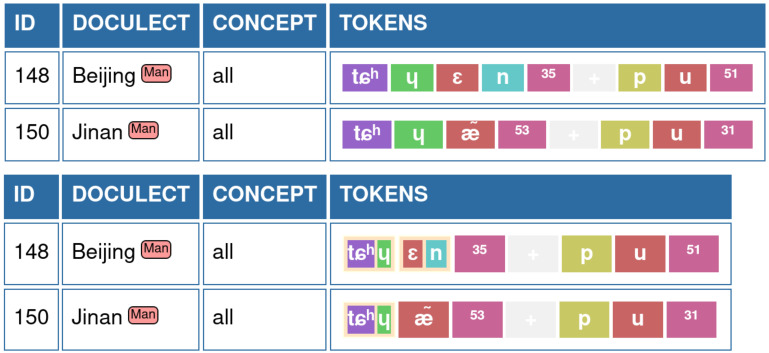
EDICTOR representation of sound sequences. **A**) shows the typical representation with colored sound classes used in previous versions. **B**) shows how grouped sounds are represented in the EDICTOR interface.

For the representation of grouped sounds, we have modified the EDICTOR tool as of Version 2.2 ( List, 2023; List, 2017, https://edictor.org). In the original EDICTOR version, sound sequences (words or morphemes) are displayed by representing individual sounds as blocks that are colored according to their underlying sound class. The notion of sound classes itself goes back to Dolgopolsky ( Dolgopolsky, 1964) and was later employed in List ( List, 2014) for the purpose of phonetic alignment and automatic cognate detection. The major idea of sound classes is to represent individual sounds that are likely to occur in regular correspondence relations in cognate words by an overarching class. Thus, sounds like [ k] and [ tʃ] were grouped into a metaclass K in Dolgopolsky’s original proposal (see List, 2014 for details). In the EDICTOR, these basic sound classes by Dolgopolsky are used to color sounds differently, in order to facilitate the comparison and alignment of words. Figure 1A provides an example on the typical representation of words for “all” in Bějīng and Jìnán Chinese (data taken from Liu *et al.*, 2007 in the version of Wu and List, 2023).

The representation of grouped sounds builds on the colored sound representation typical for the EDICTOR but assigns grouped sounds to the same square. As a result, grouped sounds occupy the same space as simple sounds, while individual background colors are used for individual sound segments, as shown in Figure 1B.

The grouping of sounds has immediate consequences for EDICTOR analyses, as the tool will treat grouped sounds as one unit. As a result, phonetic alignments are greatly facilitated and the search for sound correspondence patterns can also include grouped sounds, which helps to deal with conditioning context in a rudimentary form that may often be sufficient to disambiguate correspondence patterns on one’s data.

### Computer-assisted grouping of sounds

Since the grouping of sounds is typically done for a specific language family with a specific analysis in mind, we do not see the possibility to create a method that would group sounds directly into evolving units. While it may be possible to design algorithms that account for an approximate grouping, we would consider it as premature to devote too much time to this problem at a stage where not enough experiments with the possibility of grouping sounds into evolving units have been carried out yet.

What we can offer already, however, are two computer-assisted approaches. The first one is a method that groups sounds based on explicit prescriptions. The second one is a new routine, implemented in EDICTOR 3 ( https://edictor.org, List & van Dam, 2024), that allows for the quick manual grouping of sounds in comparative wordlists.

Our first method, which is implemented in LinSe, a Python package for sequence manipulation in comparative linguistics ( https://pypi.org/project/linse, Forkel & List, 2024), makes use of the technique of segment grouping by conversion tables that was prominently introduced as one of the major aspects of Orthography Profiles, as they were described in Moran and Cysouw (2018). The basic idea of this sequence conversion technique is to provide a replacement table that converts one sequence (e.g. written in one specific orthography) into another sequence (e.g. written in yet another orthography) while at the same time providing a segmentation of the originally unsegmented strings into distinct units. As an example, consider Table 2. On the left, there is a simple replacement table that will convert a sequence like *tian* into a segmented sequence [ tʰ j ɛ n], and a sequence *tiang* into a sequence [ tʰ j a ŋ], accordingly. All we have to do in order to apply a secondary grouping of the sounds is to define an additional replacement table for the already segmented and converted sound sequences. This is shown on the right in Table Table 2, where we represent spaces in the original sequence by underscores and replace underscores in isolation with an empty string (indicated by NULL). When applying this profile to [ tʰ j ɛ n] and [ tʰ j a ŋ], respectively, it will yield the desired grouping of sounds as [ tʰ.j ɛ.n] and [ tʰ.j a.ŋ].

**Table 2.** Using orthography profiles to segment words and convert from one transcription to another (left table) and to group sounds (right table).

Grapheme	IPA		Grapheme	GroupedSound
t	tʰ		tʰ_j	tʰ.j
i	j		ɛ_n	ɛ.n
an	ɛ n		a_ŋ	a.ŋ
ang	a ŋ		_	NULL

We illustrate this procedure by supplementing this study with a small Python script that can be used to extract grouped sounds (as annotated manually) from a wordlist and then construct an orthography profile to apply the groupings to additional datasets. In this way, users wishing to group sounds in their data can first annotate parts of their data and later apply this annotation automatically to the rest of their collection. We illustrate the suitability of this approach by applying our package to a recently standardized dataset of Karenic languages ( Luangthongkum, 2019, standardized in Luangthongkum, 2023, curated on GitHub at https://github.com/lexibank/luangthongkumkaren, Version 1.0), in which we carried out a manual grouping of all sounds in the data.

Our second method expands the functionality of EDICTOR in its most recent version, EDICTOR 3, ( List & van Dam, 2024) by allowing users to group sounds into units interactively when editing data in the main panel of the tool. In order to group sounds into evolving units, users simply have to press the CTRL key and do a right mouse click on a particular sound. As a result, the sound will be merged with the sound following it. A little button will appear to the right of the sound, allowing uses to ungroup the sounds by pressing CTRL and the right mouse button another time. 

## Examples

### Grouping and ungrouping sounds in a Karenic wordlist

In order to illustrate how grouping and ungrouping of sounds can be done in an automated way, we wrote a small Python script that starts from a dataset in which sounds have been manually grouped before. From this dataset, we create an orthography profile that is capable of grouping ungrouped sounds by extracting all graphemes from the original data (including grouped sounds) and replacing our grouping character (the dot) by a new segmentation symbol (an underscore in our case). This profile is illustrated in Table 3. With such a profile, we can convert a sequence in which sounds have not been previously grouped into both a grouped and an ungrouped representation, simply depending on the output to which we convert the previously matched sequence. Thus, if one starts from a sequence ”t a m”, we would first convert the whitespace separating sounds from each other, by the underscore. In a second step, the sequence “t_a_m” would be segmented into the three segments t, _, and a_m. These three segments could not be converted to “t” → ”t”, ”_” → ”NULL”, ”a_m” → ”a m” or ”t” → ”t”, ”_” → ”NULL”, ”a_m” → ”a.m”, respectively. This principle of converting into two representations is very simple and straightforward. But it is very useful when working with datasets where one wants to handle two segmentations at the same time.

**Table 3.** Small excerpt of our Karenic orthography profile that represents sounds in grouped and plain form.

Graphemes	Grouped	Plain	Frequency
t	t	t	99
a_m	a.m	a m	16
ə_m	ə.m	ə m	15
p_r	p.r	p r	18
o_ʔ	o.ʔ	o ʔ	21
_	NULL	NULL	—

In order to make sure that the conversion indeed yields the expected output, we test our segmentations by applying them to the whole dataset, for which the grouped sounds profile was automatically created and can show that the conversion from the ungrouped sounds back to the grouped sounds works without a single error, accounting for all sound groupings that we applied to the data manually before. The code and the data that we used for this experiment is provided as part of the supplementary material along with all information necessary to replicate the experiment.

### Grouping sounds in the comparison of Mataguayan languages

**Figure 2.**
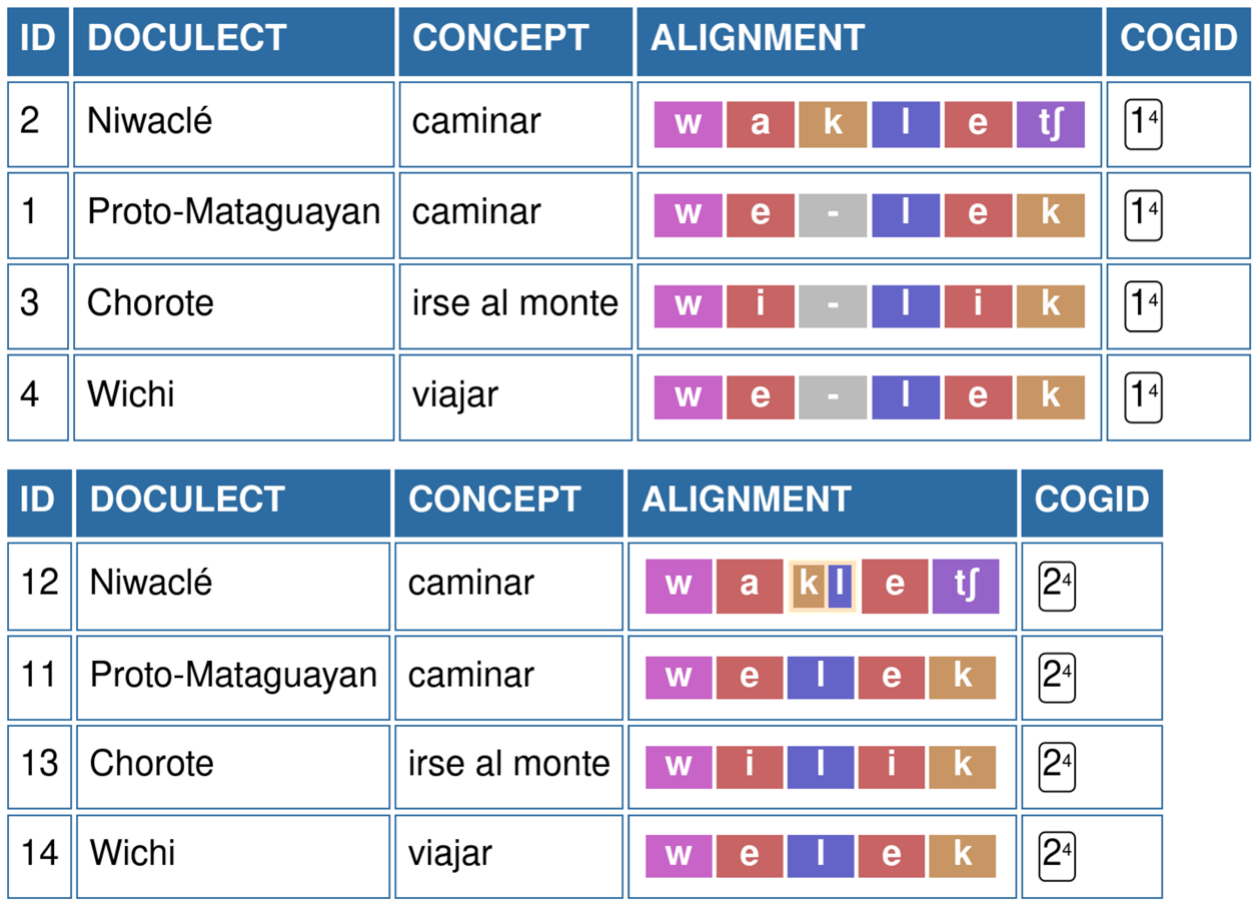
EDICTOR representation of non-grouped and regrouped sound in Mataguayan languages. Top: Segments [ k] and [ l] are treated as individual segments; Bottom: Regrouping of sound as [ k.l].

Benefits of sound grouping can also be observed when comparing languages with articulatory complex sounds, such as the case of Nivaclé, one of the four Mataguayan languages spoken in the South American Gran Chaco region. Here we consider examples coming from a dataset designed for exploring ancestral relationship in two South American language families, namely Guaycuruan and Mataguayan. Viegas Barros (2013) provides a list of (135) manually annotated cognate sets that we retro-standarized for computer-assisted analysis. Within the Mataguayan group, Nivaclé has the typologically unusual sound [ kl], which corresponds to a complex sound with a voiceless velar onset phase released into a lateral approximant ( Gutierrez, 2019:49). Figure 2 illustrates the alignment of segments for the cognate set WALK, when edited in the EDICTOR tool. In the alignment on the top, we treat the sequence [ k l] as two distinct sounds, which results in an alignment that suggests that the sound [ k] has been gained by some sound change processes from Proto-Mataguayan to Nivaclé. When grouping both [ k] and [ l] into one unit [ k.l], we receive a much more organic alignment, in which we can propose a specific sound change from Proto-Mataguayan * *l* to Nivaclé *kl*. While the specific conditions of this sound change will still need to be explained by comparative linguists (as far as we can see from the data, the pattern seems to be regular, occurring in at least 5 instances in the dataset by Viegas Barros), the resulting alignment is much more in line with both synchronic and diachronic analyses of Nivaclé in specific and Mataguayan languages in general.

### Grouping sounds in alignments of Quechuan languages

In the Quechua language family, a main criterion for distinguishing the Central Quechua group from the other branches of the family is the elision of [ j] in the sequences * *aja*, giving rise to a large vowel [ aː] ( Adelaar, 1984; Cerrón-Palomino, 2003). This change is attested both in the lexical and the morphological domain. In another variety of Quechua, Santiagueño, the same process occurs with * *awa*, independently of the aforementioned subgroup.

**Figure 3.**
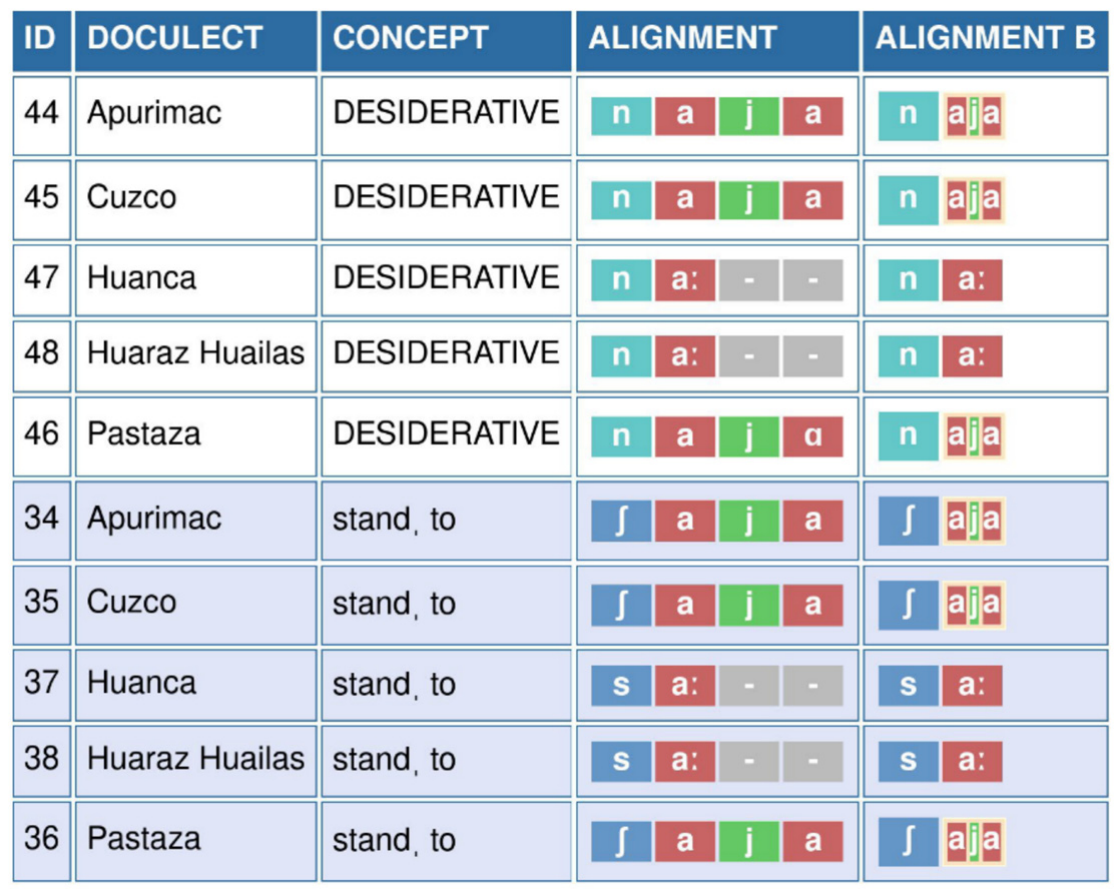
EDICTOR representation of grouping [ a j a] as [ aja] in five Quechuan varieties across two cognate sets. As can be seen from the representation, the grouping of the sounds in the column Alignment B reveals the regular nature of the correspondence.

We illustrate this change in the publicly available CrossAndean dataset ( Blum *et al.*, 2023, curated on GitHub at https://github.com/lexibank/crossandean). Figure 3 shows the annotations for two cognate sets, the lexical concept TO STAND and the DESIDERATIVE morpheme in five varieties. In both cases, we can observe that the sequence [ a.j.a] in the Quechua of Apurímac, Cuzco, and Pastaza corresponds to [ aː] in the varieties of Huanca and Huaraz-Huailas. In order to represent this change, it is necessary to group all three sounds of the sequence * *aja*. If this were not done, [ aː] would be treated as corresponding to [ a] in the sequence, while the other two sounds would need to be filled with gaps.

## Discussion and outlook

In this brief report, we have illustrated a seemingly small change to existing resources on historical language comparison. By proposing a modified annotation format and showing how it can be integrated into existing resources, we offer a solution for the problem resulting from a strict segmentation of words into speech sounds in computer-assisted approaches to comparative linguistics. Although small, however, we consider the proposal as important, since it addresses an important problem that has so far been disregarded in formal approaches in historical linguistics. Our solution of grouping sounds that were previously rigorously segmented and properly transcribed in standard phonetic transcriptions, we offer a flexible compromise that allows us to adhere to common standards (such as the International Phonetic Alphabet) while at the same time allowing for much more flexibility when carrying out phonetic alignment analyses.

## Data Availability

Zenodo: Underlying data for: Grouping sounds into evolving units for the purpose of historical language comparison. https://doi.org/10.5281/zenodo.10080690 ( List, 2024b) Data are available under the terms of the Creative Commons Attribution 4.0 International license (CC-BY 4.0)

## Software availability

Source code available from: https://github.com/calc-project/grouping-sounds/releases/tag/v1.1

Archived source code at time of publication: https://doi.org/10.5281/zenodo.10080690 ( List, 2024b)

License: CC-BY-4.0
