# Supplementary material for: Grouping sounds into evolving units for the purpose of historical language comparison
Source: Open Res Eur. 2024 Aug 20;4:31. Originally published 2024 Feb 19. [Version 2] doi: 10.12688/openreseurope.16839.2 (PMC11196928; doi:10.12688/openreseurope.16839.2)
Supplement: Peer review history

# Reviewer report: Reviewer response for version 2

**DOI:** 10.21956/openreseurope.19876.r48052

**Published:** 2025-01-02

**Authors:** Wei Zhao

**Affiliations:** 1 University of Aberdeen, Aberdeen, Scotland, UK

The idea looks interesting, but I fear experimentation is not rigorously designed. For instance, dataset details are missing - how many words are contained in the Karenic dataset, how was this dataset constructed, and what is the diversity of the manual sound grouping results? Without such information, I will be puzzled by the argument that the conversation from ungrouped sounds back to the grouped sounds works without a single error - how reliable this argument is? This issue also applies to other datasets, where quantitative experimentation seems missing. Furthermore, baseline approaches are missing, it is crucial to statistically demonstrate your approach by comparing it against baselines on the investigated datasets. Additionally, are there any limitations of your approach?

Is the work clearly and accurately presented and does it cite the current literature?

Yes

If applicable, is the statistical analysis and its interpretation appropriate?

Partly

Are the conclusions drawn adequately supported by the results?

Partly

Are all the source data underlying the results available to ensure full reproducibility?

Yes

Is the study design appropriate and is the work technically sound?

Partly

Reviewer Expertise:

computational modeling of semantics, lexical semantics, (partly) sound change.

I confirm that I have read this submission and believe that I have an appropriate level of expertise to confirm that it is of an acceptable scientific standard, however I have significant reservations, as outlined above.

# Response

**Published:** 2025-01-02

**Authors:** Johann-Mattis List

**Affiliations:** University of Passau, Germany

We thank the reviewer for this interesting report. We think, however, that we can easily answer the critics without writing a new version of the article.

 First, all data and counts are available openly, published with open archives, where everybody can check the code and all details without a problem. Second, the fact that the conversion works without flaws in both directions is also due to the fact that we use a rule-based approach for a problem that can be solved directly.

 The expectation, that algorithms never hit 100% on individual datasets is only true for problems that cannot be solved entirely. If we designed a calculator, for example, the expectation would be that it knows to add and to subtrat without any errors in a certain numeric range. The same holds for the approach that we present on the grouping of sounds. We hope that this clarifies why we would not modify our study based on this review. But we express our gratitude to the reviewer for taking the time to assess our approach.

# Reviewer report: Reviewer response for version 2

**DOI:** 10.21956/openreseurope.19876.r48055

**Published:** 2024-12-20

**Authors:** Stephen Bothwell

**Affiliations:** 1 Department of Computer Science and Engineering, University of Notre Dame, Notre Dame, Indiana, USA

This article tackles an important issue in the study of sound in computational historical linguistics (CHL, henceforth): the segmentation of phones in a sequence. Due to the limitations of phonetic notation, information about interactions among sequential phones may be lost or left to inference. These limitations cause a disconnect between computational and traditional studies of historical linguistics.

 To remedy this disconnect, the authors propose a notation for grouping adjacent phones together, hoping to better reflect the evolution of language in sequence alignment. In addition, the authors also augment two pieces of software to promote the use of their notation: LinSe, a Python library, and EDICTOR 3.0, a web interface. They exhibit the capabilities of their software and the utility of their notation in producing more natural evolutionary sequence alignments through Chinese, Karenic, Mataguayan, and Quechuan languages.

 Overall, this paper is quite well-written and conveys clearly, both in plain language and in a more technical manner, the contribution and implementation of evolving sound units. The paper argues that its contribution is small and straightforward, and I do not think it has overstated its claims. In addition, I feel that exploring the limits of phonetic notation for CHL is a critical and relevant topic for advancing the use of computational tools in historical linguistic studies. The array of examples shows well that allowing many-to-many alignments between phones in distinct phonetic sequences permits more sensible analyses for explaining the relationships between sounds in related lects.

 To me, the crux of this work is a matter of perspective. Evolving sound units provide a new perspective for phonetic notation in the context of CHL, making explicit which sounds change together. The usefulness of this notation has been made clear to me from an analytical perspective, but from a practical point of view, it is not yet evident whether what may be called a prescriptive tokenization of phonetic sequences may be applied further in the field. If some additional practical grounding could be provided for this notation (perhaps in terms of computational efficiency, learnability, or interpretability), I feel that its overall contribution would be stronger. However, I again think that this work has not overstated its accomplishments and could see an exploration of its computational applications in future work.

 On another note, while I found that applying the authors’ notation in the EDICTOR 3.0 software is very straightforward, I felt that LinSe was a bit more indirect. The publication on LinSe indicates where to find the Orthography Profiles that are central to the segmentation algorithm; it also provides an example application of Orthography Profiles in LinSe with the SegmentGrouper class. However, a lack of documentation for the library may hinder some users in applying this notation. Adding documentation aside from the paper may help users employ the library for the purpose intended in this article.

 Finally, aside from the above comments, I wanted to note a few places in the current manuscript where edits could be warranted.

 (p4, first full paragraph): “… computational approaches have difficulties to recognize valid sounds …” The phrasing here with the infinitive seems somewhat awkward to me. Perhaps “… computational approaches have difficulties in recognizing valid sounds …” would work better?

 (p4, fifth full paragraph): “… with our new alignment where we cluster sounds historically likely relevant units.” There seems to be a preposition missing here; perhaps change to “… with our new alignment where we cluster sounds into historically likely relevant units.”

 I am not sure that edits to the “Amendments” section matter for later versions of the article, but I have included one in case they do.

 (Amendments): “The code has also be modified, using a new software package, that we have created for this purpose.” In this sentence, I believe “be” should be “been”; in addition, the latter part of the sentence (“using … purpose”) is somewhat confusing to me, as it could imply (incorrectly, I believe) that the software package is what modified the code. I believe that the authors are trying to say something to the effect of “We have created a new software package and have modified our code to integrate this package.”

Is the work clearly and accurately presented and does it cite the current literature?

Yes

If applicable, is the statistical analysis and its interpretation appropriate?

Not applicable

Are the conclusions drawn adequately supported by the results?

Yes

Are all the source data underlying the results available to ensure full reproducibility?

Yes

Is the study design appropriate and is the work technically sound?

Yes

Reviewer Expertise:

natural language processing, computational historical linguistics, digital humanities, language modeling

I confirm that I have read this submission and believe that I have an appropriate level of expertise to confirm that it is of an acceptable scientific standard.

# Response

**Published:** 2024-12-20

**Authors:** Johann-Mattis List

**Affiliations:** University of Passau, Germany

We want to express our gratitude to the reviewer. If we manage to come up with an updated version in the next year, we will gladly take the suggestions for amendmends into account and correct the errors mentioned. As to the documentation questions of LinSe, we hope to further enhance the library in the future as well, but we cannot promise yet when this would be done.

# Reviewer report: Reviewer response for version 2

**DOI:** 10.21956/openreseurope.19876.r47615

**Published:** 2024-12-17

**Authors:** Ander Egurtzegi

**Affiliations:** 1 Centre National de la Recherche Scientifique, CNRS, Paris, France

This brief report presents an improvement to sound sequence representations in computational models of phonological comparison within cognate sets. The new idea involves grouping individual sound segments into sequences of sound that have been observed to evolve as a unit. The report is clear, concise, well written (I only found a typo at the end of the “Amendments” section), and very easy to follow.

 This approach seems to be especially useful to historical linguists working on South-East Asian languages, but it can also be useful to model sound changes where a sound influences an adjacent sound before it is lost (as in the development of nasal vowels from VN sequences or tonogenesis), which are widespread in the world’s languages inasmuch they stem from cross-linguistically recurring coarticulatory patterns. It is also likely that this method can account for cases of local metathesis (which is the most likely to be regular) much better than the previous one. Nonetheless, this approach would not help model coarticulation-based developments that occur at a distance (e.g. umlaut). There might be a (maybe not so simple and visually elegant) way to accommodate to these as well, which would be a welcome future addition. In any case, the alignment resulting from the improvement described here clearly improves the previously available one for the examples discussed in the report.

 In addition, the implementation of the features seems easy to use and user-friendly. The examples used as an illustration come with the link to the open-access database used for each of them. The links to the repositories with the relevant data and source code are also included in the report. All this greatly facilitates the replication of the examples for the reader, which is very appreciated.

 However, this approach can result in the oversimplification of historical change by conflating sequences of independent changes affecting sounds in the same phonological context, as illustrated by the authors with an example from German sibilant retraction. In addition, segment grouping can bias analysis by means of adding a priori designated clusters that may increase the effect of confirmation bias. Of course, this is not a problem of the software in itself, but it is something the analyst needs to keep in mind when performing this kind of analysis (or basically any other, for that matter).

 In short, this brief report presents a useful addition to the toolbox for computational analysis of historical phonological data. An interested reader can easily understand the use and possibilities of the new feature of the EDICTOR software by reading it, as well as finding the relevant links to the data and source code at the end of the report.

Is the work clearly and accurately presented and does it cite the current literature?

Yes

If applicable, is the statistical analysis and its interpretation appropriate?

Not applicable

Are the conclusions drawn adequately supported by the results?

Yes

Are all the source data underlying the results available to ensure full reproducibility?

Yes

Is the study design appropriate and is the work technically sound?

Yes

Reviewer Expertise:

Historical phonology, phonetics, sound change.

I confirm that I have read this submission and believe that I have an appropriate level of expertise to confirm that it is of an acceptable scientific standard.

# Response

**Published:** 2024-12-17

**Authors:** Johann-Mattis List

**Affiliations:** University of Passau, Germany

We express our gratitude to the reviewer for this positive review. We hope that future research can prove the usefulness of our new coding scheme.

# Reviewer report: Reviewer response for version 2

**DOI:** 10.21956/openreseurope.19876.r47618

**Published:** 2024-12-13

**Authors:** Susanto Susanto

**Affiliations:** 1 Universitas Bandar Lampung, Bandar Lampung, Lampung, Indonesia

The article presents a novel approach to improving sound sequence representations in computational historical linguistics. It aims to address the limitations of traditional rigid sound segmentation by allowing for the grouping of sounds into evolving units. This flexibility is intended to enhance the analysis of language-specific sound laws and improve the alignment of phonetic data across different languages.

 The introduction of evolving sound units is a significant advancement in the field, providing a more nuanced understanding of sound changes over time. The accompanying software library adds practical value, enabling researchers to convert their data between traditional and evolving sound unit formats. Concrete examples, particularly from Chinese and Mataguayan languages, effectively illustrate the benefits of the proposed method.

 The article mentions a software library but lacks detailed documentation on its implementation. Including a section on how to use the software, along with examples of input and output data, would make it more accessible to researchers unfamiliar with the tools.

 A candid discussion of potential limitations and challenges associated with the new approach would provide a balanced view. For instance, are there specific language families where this method may not yield accurate results?

 The article offers a valuable contribution to the field of historical linguistics by addressing a critical issue in sound segmentation. By enhancing software documentation, and expanding the discussion of potential limitations and challenges, the authors can significantly improve the scientific rigor and overall impact of their work. The proposed methodology has the potential to advance the study of language evolution and sound change, provided it is presented with the necessary clarity and detail.

Is the work clearly and accurately presented and does it cite the current literature?

Yes

If applicable, is the statistical analysis and its interpretation appropriate?

Not applicable

Are the conclusions drawn adequately supported by the results?

Yes

Are all the source data underlying the results available to ensure full reproducibility?

Yes

Is the study design appropriate and is the work technically sound?

Partly

Reviewer Expertise:

phonetics, phonology, computational linguistics

I confirm that I have read this submission and believe that I have an appropriate level of expertise to confirm that it is of an acceptable scientific standard, however I have significant reservations, as outlined above.

# Response

**Published:** 2024-12-16

**Authors:** Johann-Mattis List

**Affiliations:** University of Passau, Germany

We express our gratitude to the reviewer for this comment. Since the article was meant as a brief report on an ongoing system of annotation, we limited examples and discussions in the article itself, hoping that future work would reveal the usefulness of the study. When writing an update later, we will demonstrate that even by now, much more documentation on the new feature is already available, specifically in the particular tools that make use of the new annotation practice.

# Reviewer report: Reviewer response for version 2

**DOI:** 10.21956/openreseurope.19876.r46347

**Published:** 2024-12-11

**Authors:** Ewan Dunbar

**Affiliations:** 1 University of Toronto, Toronto, Ontario, Canada

This brief report describes a modification to existing tools for doing historical linguistics, namely, a mechanism by which segments can be grouped together for the purposes of alignment. The obvious applications of this are complex segments (diphthongs and affricates), as these are known to behave differently than sequences, both synchronically and historically, but the paper demonstrates the effect of this tool on a number of other cases, including a case where a glide is elided and the surrounding two syllables coalesce into a single long vowel (note that the paper says "large vowel" - I believe that "long" was meant).

 The major limitation of this paper is its very narrow scope. To be perfectly transparent, I am not a historical linguist, and, therefore, some of the things that may be implicit, I am not able to guess. For example, while it is very clear to me how historical analyses could benefit from grouping a diphthong into a single complex segment, it would have been useful to demonstrate what exactly the tangible downstream consequences are for computational linguistics, as referred to in the first paragraph. The paper repeatedly refers to figures showing alignments and points to the fact that they are "more in line with" traditional analyses of the languages in question. Perhaps visually, but the utility of grouping depends entirely on the analysis being done and the question being asked. If my research question required me to *identify* cases where two vowels in adjacent syllables coalesce into one, it would be counterproductive to turn the two-syllable sequence into a single unit. Clearly, this is intentional: the grouping is manual, and so it is very obviously intended to be used by researchers who know what they are doing and why! However, without more concrete use cases, it is difficult for me to fully appreciate the degree to which this tool is really needed, or, rather, whether it represents a minor convenience. While this is a brief report and is meant to be limited in scope, I believe it would greatly benefit from more concrete discussion about specific analysis problems that would benefit from the technique proposed.

 Nevertheless, the paper competently and clearly presents the technique, such as it is, and is likely to be useful to the target audience.

Is the work clearly and accurately presented and does it cite the current literature?

Yes

If applicable, is the statistical analysis and its interpretation appropriate?

Not applicable

Are the conclusions drawn adequately supported by the results?

Partly

Are all the source data underlying the results available to ensure full reproducibility?

Yes

Is the study design appropriate and is the work technically sound?

Yes

Reviewer Expertise:

Computational linguistics; speech processing; phonology

I confirm that I have read this submission and believe that I have an appropriate level of expertise to confirm that it is of an acceptable scientific standard, however I have significant reservations, as outlined above.

# Response

**Published:** 2024-12-11

**Authors:** Johann-Mattis List

**Affiliations:** University of Passau, Germany

We thank the reviewer for this very useful review on our little study on the grouping of sounds. Whether we will be able to account for the points raised, i.e., to provide detailed examples of why it may be useful to group sounds or to not group sounds, however, is something that we won't be able to answer to soon, since studies are still ongoing, and the annotation itself is tedious. What we can say for now, however, and we may well add this at a later stage to an updated version of the study, is that linguists are in fact already using versions of grouped sounds for particular language families (mostly South-East Asian languages). Thus, the tool as such may not need to prove its usefulness, we would argue, but rather makes sure that our computer-assisted approaches come closer to the needs of traditional analyses. If the reviewer would find an extended investigation of such differences in analysis useful, we'd gladly consider to update the report soon.

# Reviewer report: Reviewer response for version 1

**DOI:** 10.21956/openreseurope.18194.r40344

**Published:** 2024-06-24

**Authors:** Jelena Prokić

**Affiliations:** 1 Leiden University, Leiden, The Netherlands

The article presents new sound sequence representation and annotation format that allows researchers to group individual sound segments into sound units. This research is a valuable contribution to the field of computational historical linguistics since it will allow researchers to go beyond current strict segment models. As shown by the authors, it is a very useful method while working with certain languages, like Nivaclé. It still remains to be seen how widely this kind of representation can or will be used while working with different languages. The conversion to sound units cannot be done automatically and requires annotated data or a list with mappings (an orthography profile). The proposed solution is useful when sound units are already determined by the researcher. Sound unit representation is a valuable addition for the sequence alignment analysis, but the application in automatic language comparison and analyses like loan detection needs to be investigated. This research can be seen as the first step toward incorporation of new sequence representation into computer aided language research. 

 The paper is clearly written and easy to follow. The only exception is a section where the authors say that 'the number of distinct speech sounds accounted for by the CLTS reference catalog is too large to reflect the linguistic reality of phonetic diversity in the languages of the world'. This sounds confusing and it should be better explained. Why is the case that the a large number of distinct sounds is not a good way to represent phonetic diversity in the languages of the world?

 The paper includes accompanying code and data and adheres to values and principles of open science.

Is the work clearly and accurately presented and does it cite the current literature?

Yes

If applicable, is the statistical analysis and its interpretation appropriate?

Not applicable

Are the conclusions drawn adequately supported by the results?

Yes

Are all the source data underlying the results available to ensure full reproducibility?

Yes

Is the study design appropriate and is the work technically sound?

Yes

Reviewer Expertise:

dialectometry, quantitative linguistics, computational linguistics

I confirm that I have read this submission and believe that I have an appropriate level of expertise to confirm that it is of an acceptable scientific standard.

# Response

**Published:** 2024-08-05

**Authors:** Johann-Mattis List

**Affiliations:** University of Passau, Germany

Thanks a lot for these very helpful comments. We have tried to enhance our revised version by adding code examples that are more transparent and also made up for some typos in our previous version, adding new references.

# Reviewer report: Reviewer response for version 1

**DOI:** 10.21956/openreseurope.18194.r40340

**Published:** 2024-05-28

**Authors:** Erich Round

**Affiliations:** 1 University of Surrey, Guildford, England, UK; 2 School of Languages and Cultures, The University of Queensland (Ringgold ID: 1974), Saint Lucia, Queensland, Australia

**Summary**

 This brief report illustrates a small but powerful change to existing resources on historical language comparison, namely allowing the grouping of individual speech sounds into larger molecules prior to alignment. This is a smart innovation which solves two connected problems: (1) that sounds may pattern in a 1:many or many:many way wrt alignment, and (2) that simply enumerating all combinations would be combinatorially unfeasible.

**Add two recent, relevant references on p3**

 - To Hartmann 2003 add Marr & Mortensen 2023[Ref 1]

 - "The problem of segmentation is also important for the level of phonetic transcription." Here, add Round 2023[Ref 2]

**Clarify one point on p4**

 "shows that it would not be feasible to try to account for all possible sound combinations", > "shows that for reasons of combinatorial explosion, it would not be feasible to try to account for all possible sound combinations"

**Suggestion to clarify a bit in the abstract:**

  "are segmented into fixed sound units which cannot be modified" > "are segmented into units of sound whose boundaries cannot be manipulated"

Is the work clearly and accurately presented and does it cite the current literature?

Partly

If applicable, is the statistical analysis and its interpretation appropriate?

Not applicable

Are the conclusions drawn adequately supported by the results?

Yes

Are all the source data underlying the results available to ensure full reproducibility?

Yes

Is the study design appropriate and is the work technically sound?

Yes

Reviewer Expertise:

Computational historical linguistics; Phonology; Morphology

I confirm that I have read this submission and believe that I have an appropriate level of expertise to confirm that it is of an acceptable scientific standard.

## References

[1] : Large-scale computerized forward reconstruction yields new perspectives in French diachronic phonology. *Diachronica* .2023;40(2) : 10.1075/dia.20027.mar 238-285 10.1075/dia.20027.mar

[2] : Canonical phonology and criterial conflicts: relating and resolving four dilemmas of phonological typology. *Linguistic Typology* .2023;27(2) : 10.1515/lingty-2022-0032 267-287 10.1515/lingty-2022-0032

# Response

**Published:** 2024-08-05

**Authors:** Johann-Mattis List

**Affiliations:** University of Passau, Germany

Thanks a lot for these insightful comments. IN our updated version, we have tried to account for all suggested changes and hope that this has been done in a satisfying way.
